# Community based peer-led TB screening intervention: an innovative approach to increase TB knowledge, presumptive case identification, and referral among sexual minority people in urban Bangladesh

**DOI:** 10.1186/s12913-023-09737-5

**Published:** 2023-07-29

**Authors:** Golam Sarwar, Shaan Muberra Khan, Samira Dishti Irfan, Mohammad Niaz Morshed Khan, Md. Masud Reza, A K M Masud Rana, Rupali Sisir Banu, Shahriar Ahmed, Sayera Banu, Sharful Islam Khan

**Affiliations:** 1 Programme for HIV and AIDS, Infectious Diseases Division, International Centre for Diarrhoeal Disease Research, Bangladesh (icddr,b), 68, Shaheed Tajuddin Ahmed Sarani Mohakhali, Dhaka, 1212 Bangladesh; 2 National Tuberculosis Control Programme (NTP), Directorate General of Health Services (DGHS), Ministry of Health and Family Welfare (MOH&FW), Dhaka, Bangladesh; 3 Programme for Emerging Infections, Infectious Diseases Division, International Centre for Diarrhoeal Disease Research, Bangladesh (icddr,b), Dhaka, Bangladesh

**Keywords:** Tuberculosis (TB), TB screening, Referral, Sexual minority people

## Abstract

**Introduction:**

One of the contributors to tuberculosis (TB) burden among vulnerable populations, such as sexual minority people, is the delay in case finding and notification. Given their socially excluded, hard-to-reach nature, community-led approaches need to be introduced to facilitate their screening of TB symptoms and their subsequent referral to TB healthcare providers. This article aimed to explore the existing challenges surrounding TB screening and referral, and the implementation facilitators and barriers of the proposed community-based TB screening model for sexual minority people in Dhaka, Bangladesh.

**Methods:**

This study followed the quasi-experimental design using mixed methods (i.e., qualitative and quantitative) approach. The study participants who were also a part of the community-led TB screening model included sexual minority people enrolled in HIV prevention interventions. In addition to quantitative inquiry, in-depth interviews were conducted on sexual minority people, focus group discussions were also conducted on them and HIV prevention service providers, and key-informant interviews were conducted on service providers, programmatic experts and TB researchers. Data were analyzed using content, contextual and thematic approaches.

**Results:**

The ‘Six Steps in Quality Intervention Development’ framework was used to guide the development of the community-based TB screening model. In Step 1 (identifying the problem), findings revealed low rates of TB screening among sexual minority people enrolled in the HIV prevention intervention. In Step 2 (identifying contextual factors for change), various individual, and programmatic factors were identified, which included low knowledge, low-risk perception, prioritization of HIV services over TB, and stigma and discrimination towards these populations. In Step 3 (deciding change mechanism), community-based screening approaches were applied, thus leading to Step 4 (delivery of change mechanism) which designed a community-based approach leveraging the peer educators of the HIV intervention. Step 5 (testing intervention) identified some barriers and ways forward for refining the intervention, such as home-based screening and use of social media. Step 6 (collecting evidence of effectiveness) revealed that the main strength was its ability to engage peer educators.

**Conclusion:**

This study indicates that a community-based peer-led TB screening approach could enhance TB screening, presumptive TB case finding and referral among these populations. Therefore, this study recommends that this approach should be incorporated to complement the existing TB program.

## Introduction

Tuberculosis (TB) was the top cause of death from a single infectious agent and the 13^th^ leading cause of death globally [1]. In 2021, an estimated total of 10.6 million new cases (range, 9.9–11.0 million) were diagnosed with TB worldwide, 6.7% of whom were people living with HIV (PLHIV). Recent global evidence indicates 187,000 TB deaths among PLHIV [1]. TB estimated incidence rate increased between 2020 and 2021, and it is worth noting that some regions face a substantial TB burden. For example, 45% of the diagnosed TB cases in 2021 originated from the World Health Organization (WHO) region of Southeast Asia. Furthermore, Bangladesh is among the eight countries in the Globe that account for two-thirds of the total TB cases [1]. Bangladesh is also one of the 30 high TB burden countries in the world with a documented incidence of 221/100,000 and an estimated mortality count of 25/100,00 people as of 2021. The estimated HIV-positive TB incidence was 0.43 per 100,000 population [1]. According to the annual report (2020) of National Tuberculosis Control Programme (NTP) in Bangladesh, a total of 2,508 PLHIV were tested for TB, of which 434 PLHIV were diagnosed as TB from 2015–2019, which is 17.3% [2]. However, there is no data available regarding the proportion of sexual minority people [i.e., males having sex with males (MSM), male sex workers (MSW) and transgender women] with HIV in Bangladesh who have TB.

In Bangladesh, the HIV prevalence is low at 0.01% among the general population but the prevalence remains relatively high among the key populations (KPs) at risk of HIV [3]. According to the size estimation of the KPs in Bangladesh (2015–16) the estimated number of MSM (including MSW) is 131,472 and TG is 10,199, with a total of 141,671 estimated sexual minority people in Bangladesh [4]. The total population in Bangladesh is 165 million [5], thus amounting the total proportion of sexual minority people in Bangladesh to 0.09%. UNAIDS/WHO estimates of at least 1% adult population in male-to-male sex makes this proportion even higher [6]. As per the Integrated Biological and Behavioural Survey (IBBS) among KPs at High Risk of HIV in Bangladesh, 2020, prevalence of HIV among female sex workers (FSW) was 0.1%, 1.7% among MSM, 0.9% among TG, and 2.4% among people who inject drugs (PWID) [3]. Therefore, the sexual minority people are disproportionately affected by the HIV burden. In this context, to contain the further spread of HIV infection among sexual minority people, there are a total of 50 HIV prevention service centers/drop-in centers (DICs) throughout 37 districts in Bangladesh. The DICs are not situated within the mainstream healthcare setup, rather they are separate community-based entities funded by donors and operated by non-government organizations (NGOs). To ensure accessibility and convenience for the sexual minority people, the DICs are typically located in the catchment areas where sexual minority people were found to be prominent.

KPs including sexual minority people, PWID and FSW are vulnerable to TB infection [7]. Specifically, hidden and hard-to-reach MSM and transgender populations have been identified as groups with elevated vulnerability to TB infection [8]. Their socio-structural challenges, such as discrimination and stigmatization, have increased their reluctance to visit healthcare facilities, thus exacerbating their risk of adverse health outcomes [9, 10]. According to UNAIDS estimates, more than half of the HIV-associated TB cases were not diagnosed or treated as of 2018 [11]. Delayed TB diagnosis increases the risk of disease transmission among close contacts [12]. In this context, evidence highlights the importance of HIV and TB service integration [13].

The HIV prevention interventions for sexual minority people in Bangladesh have provisions for verbally screening the program participants, who are enrolled in the DIC mother list, for TB by the medical assistant (MA) on a regular basis irrespective of presence of any TB symptom whenever they visit the DICs for clinical services [i.e., sexually transmitted infection (STI) management, HIV testing services (HTS), counseling services, etc.]. Therefore, presenting symptoms of TB is not a criterion for TB screening at DIC. Yet, they need to be present at the DIC to uptake these services. Therefore, an in-person visit of sexual minority people to the DIC is crucial for ensuring verbal TB screening among these groups. Not all the sexual minority people who are enrolled in the DIC visit regularly. Rather, they mostly come for health problems. Therefore, only those who visit the DIC for any service are screened for TB. Programmatic data also revealed that the sexual minority people who are listed under specific DIC and visit DIC for any health service come under TB screening offered by the DIC, which constitutes a small proportion. For example, programmatic data indicated that merely 26% of the enlisted sexual minority people at Jatrabari DIC (265 out of 1,002) and 16% at Darus Salam DIC (146 out of 912) received verbal TB screening within the last six months (July to December 2018).

According to programmatic evidence, some sexual minority people in the community presented symptoms such as coughing for over two weeks. Based on the existing facility-based TB screening procedure in the DIC, any presumptive TB case found was referred to the DOTS centers of government or non-government facilities through the accompanied referral approach. Yet, as previously mentioned, few presumptive cases were found due to the comparatively low attendance of sexual minority people at the DICs on a regular basis. Although the National TB guideline (2021) in Bangladesh outlined the importance of prioritizing verbal TB screening for vulnerable, marginalized populations, including KPs [14], due to socio-structural challenges, it is hard to achieve coverage of these populations. Moreover, as per the National TB guideline (2021), all PLHIV should be screened for TB using rapid diagnostic tool (Xpert MTB/RIF) for at least once a year [14].

Using the 'Six Steps in Quality Intervention Development (6SQuID)’ analytical framework [15], this article presents qualitative findings from the baseline, and intervention phase of a community-based TB screening intervention study with an aim to (1) identify the gaps and challenges in the existing DIC based TB screening process which need to be addressed and (2) the scope of community-based TB screening intervention for increasing knowledge on TB (cause, transmission and prevention), presumptive case identification and referral linkage for treatment of TB among the sexual minority people in Bangladesh.

## Methods

### Study design and setting

The study design, setting, research plan, and timeframe are delineated in the published protocol [16]. The study was conducted from January 2019 to November 2020. To address the objectives of the study, using mixed methods (i.e., qualitative and quantitative) approach, this study followed a quasi-experimental design [17] with mother-listed sexual minority people in selected DICs, under the framework of operational research. The quasi-experimental study, like its true-experimental counterpart, has the ability to generate evidence about the effectiveness of an intervention [17]. In some circumstances, it is not always possible to randomize groups due to the smaller availability of sample sizes, ethical considerations, logistical challenges and social issues, therefore the quasi-experimental design is deemed a viable alternative [17, 18]. The study was conducted in two DICs: *Jatrabari* (intervention site, represents the Southern part of Dhaka city) and *Darus Salam* (comparison site, located in Western part of Dhaka city). These purposively selected DICs were operated by a local non-government organization (NGO), *Bandhu* through the management of icddr,b under the financial support of the Global Fund since 2010. We selected them as the intervention and comparison DICs due to similar programmatic attributes (i.e., duration of service operation, the concentration of HIV positive cases, number of sexual minority people enlisted etc.) and service provision (i.e., availability of HIV prevention services).

### Conceptual framework

We followed the ‘Six Steps in Quality Intervention Development (6SQuID)’ model to guide the design of the intervention [15]. This framework highlights six critical steps, namely: (1) “defining and understanding the problem and its underlying causes”; (2) “identifying malleable causal or contextual factors with greatest scope for change”; (3) “deciding how to facilitate the change mechanism”; (4) “identifying ways to deliver the change mechanism”; (5) “testing and refining the intervention”; and (6) “collecting sufficient evidence of effectiveness to proceed to rigorous evaluation of the intervention”.

### Data collection methods, study population and sampling

The findings presented in this article are based on qualitative data, which was collected during the baseline and intervention phases. We adopted multiple qualitative data collection methods including key-informant interviews (KIIs), in-depth interviews (IDIs), and focus group discussions (FGDs) with the study participants to triangulate the findings. We sampled the study participants purposively for the entire data collection to encapsulate perspectives from various stakeholders. We conducted 25 KIIs with DIC managers, medical assistants and front-line responders on the field [peer educators (PEs) and outreach supervisors] at DICs, program managers working with key-populations, NGO and public healthcare practitioners and TB researchers. We conducted one FGD with medical assistants, who provided clinical services to the sexual minority people at the DICs to gather diversified views on the existing TB screening approach, its barriers and possible ways out. We contacted the participants beforehand to ensure their availability and conducted interviews at their preferred venues and schedule.

To undertake this qualitative investigation, we conducted 32 IDIs, participants of which were sexual minority people enlisted under either *Jatrabari* DIC or *Darus Salam* DIC; who were ≥ 18 years of age and screened for TB by the medical assistant at least once within the last year prior to the study. Additionally, we arranged two FGDs with sexual minority people and three FGDs with peer educators to triangulate findings from IDIs and KIIs regarding existing challenges of the current verbal TB screening process, possible intervention strategies and delivery mechanisms.

Though the article is predominantly focused on findings based on qualitative data, in an attempt to triangulate the qualitative findings, we also incorporated quantitative analysis from the intervention and comparison sites regarding TB screening, presumptive case identification and testing (TB care cascade) variables. Baseline data on the number of TB screening, presumptive case identification, and TB testing of both intervention and comparison sites was collected from programmatic data of existing DIC-based TB screening approach. Whereas, the end-line data of intervention site was collected from intervention data and comparison site from programmatic data of existing DIC-based TB screening approach. A few of these quantitative findings have been converged with the qualitative analysis through the results section of this paper.

### Development process of community-based TB screening intervention

**Fig. 1:**
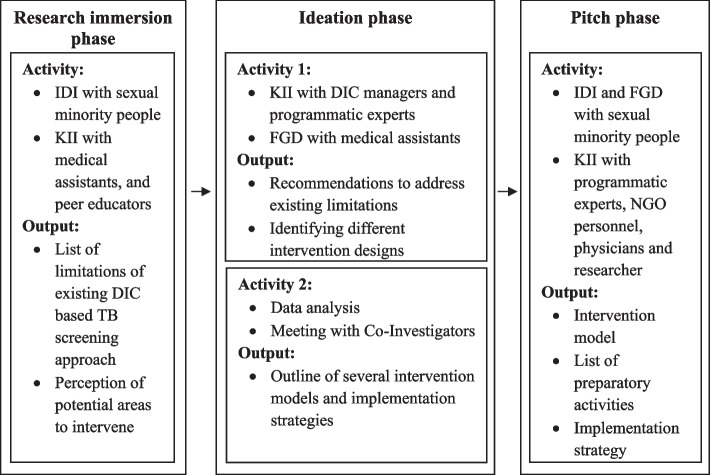
Development process of community-based TB screening intervention

We divided our data collection into three stages (i.e., research immersion stage, ideation stage, and pitch stage) to allow time for familiarizing and organizing the collected data, then relate it to the objective of the community-based TB screening intervention development (Fig. 1). First, we conducted KIIs with DIC staff (medical assistant, PE and outreach supervisor) and IDIs with sexual minority people to understand the existing DIC-based TB screening approach, challenges and identify the focus areas for intervention (i.e., internalized stigma, programmatic priority, staff capacity, etc.) in the research immersion stage.

Following that, in the ideation stage, we shared these challenges with the other key-informants (DIC managers, program managers working with key-populations, NGO and public healthcare practitioners and TB researchers) to understand their perspectives and recommendations for addressing these challenges within the current program structure/setting and culture. FGD with medical assistants was conducted along with KIIs. The ideation stage was aimed at generating different intervention design concepts. We then conceptualized possible intervention approaches to increase the: (i) number of TB screening and (ii) TB knowledge (i.e. cause, mode of transmission, prevention, diagnosis, treatment, service availability, etc.) among sexual minority people. We organized a series of meetings with Co-Investigators (i.e., experienced researchers working with KPs and experts in TB research) to deliver recommended intervention approaches from our data and seek their feedback to optimize the delivery of our proposed intervention.

Following the ideation stage, various intervention components emerged, which were presented to sexual minority people during IDIs and key informants during KIIs in the pitch stage. To elicit the societal and normative views among the community about the barriers of TB screening, one separate FGD was also conducted with sexual minority participants. FGDs sometimes create opportunities to reveal normative information, shared views or diverse views which may be valuable for exploring required information pertaining to the objective of the study. This approach was applied to complement the in-depth perspectives elicited from the IDIs. This qualitative exploration thus allowed us to understand the feasibility of each intervention component, possible field implementation challenges and best-practice intervention measures for improving these outcomes (i.e., TB screening, presumptive TB case referral, TB case detection, knowledge of TB among sexual minority people).

### Data analysis

All interviews were conducted in Bengali. With the permission of participants, interviews and FGDs were recorded using digital voice recorders. In addition, data collection and data analysis were integrated as qualitative data collection and analysis is an ongoing and reflexive process [19, 20]. This helped to identify the point of data saturation. A four-member data management team, consisting of experienced qualitative researchers, reviewed the transcripts, data familiarization and organizing relevant theme-based codes in a way that minimized bias and represented participant views. The team followed the framework analysis approach [21] where the codes were formulated based on the research questions. Emerging codes were identified from the familiarization and assigned to the relevant theme. We charted the codes under themes and organized the themes after reaching a consensus. ATLAS.ti (version 5.2) was used to facilitate data management and analysis. We reviewed the coded data multiple times to ensure the inclusion and reflection of the participants' thoughts and perspectives, and improve the credibility of our interpretation. During analysis, atypical or diverse data were not ignored, rather they were further explored, analyzed and presented as findings. The results were summarized and presented according to the context, and some data was presented verbatim to validate or reflect important views and ideas.

For the quantitative data, statistical analysis was performed using Stata version 13.0 (Stata Corp Inc., College Station, Texas, USA). Differences of TB care cascade in each intervention and comparison sites between pre-intervention and intervention period, and difference between intervention and comparison sites during intervention period was determined by χ2 test (*p* < 0.05 was considered as a significant level during comparing of TB care cascade).

### Ethical consideration

The protocol was reviewed and approved by icddr,b’s Institutional Review Board (IRB), Research Review Committee (RRC) and Ethical Review Committee (ERC). We obtained informed written and or/verbal consent from all study participants. We explained the study objectives, activities, anonymity and confidentiality to the participants and assured them of their rights to withdraw at any given time.

## Findings

The findings section is systematically categorized according to the ‘Six Steps in Quality Intervention Development (6SQuID)’ model describing the problem, underlying causal or contextual factors, change mechanism, intervention delivery approach, refinement, and evidence of the effectiveness of the intervention in a stepwise manner (Table 1).

**Table 1:** ‘Six Steps in Quality Intervention Development (6SQuID)’

Steps no	Issue	Description	
1	Defining and understanding the problem and its underlying causes	Low rate of TB screening through existing DIC-based TB screening approach, therefore low presumptive TB case identification and TB case detection	
2	Identifying which causal or contextual factors are malleable and have the greatest scope for change	Individual factors	• Low knowledge, awareness and perceived susceptibility of TB among the sexual minority people • Low perceived severity of TB and incomplete treatment • Casual attitude of sexual minority people towards health and disease • Floating modality of the sexual minority people
		Programmatic factors	• Only DIC based TB screening • Prioritization of HIV/STI services over TB • Limited capacity building initiatives on TB • Working hour of DIC: barrier for employed sexual minority people
3	Deciding how to bring about the change mechanism	• Community based TB screening • Selecting actor of community-based TB screening	
4	Identifying how to deliver the change mechanism	• Screening of sexual minority people at spot/residence/ deras maintaining confidentiality • Accompanied referral of presumptive TB cases, clinical investigation, and follow up • Specific TB training for peer educators • TB related information dissemination • Sensitization of sexual minority community for eliminating anticipatory discrimination at public health facilities • Sensitization at TB DOTS centers	
5	Testing and refining the intervention	• TB screening at home/workplace/residence of *hijra* leader • TB screening at social gatherings • Communication through social media/mobile phone	
6	Collecting sufficient evidence of effectiveness to proceed to rigorous evaluation of the intervention	• Increased verbal TB screening, presumptive cases identification and referrals • Increased TB-related knowledge among staff and participants • Changes in attitudes towards the TB DOTS services • Debunking misconception about TB	

### Step 1: Defining and understanding the problem and its underlying causes

In the existing DIC-based TB screening approach, when the sexual minority people come to the DIC presenting any health complaint, the medical assistant (MA) conducts a preliminary verbal TB screening using the prescribed verbal TB screening tool [22]. This includes a series of questions about potential TB-related symptoms. Thus, the MA is responsible for performing verbal TB screenings. According to the DIC guideline, if the MA suspects any presumptive TB case, the patient is referred to the nearest NTP operated Directly Observed Treatment, Short-course (DOTS) center. The MA from the comparison site relayed his role in the screening process in the following way:

“Whenever the beneficiaries come to me for clinical services, I inform them, “We also work with TB. I will ask you some questions, if you have these symptoms or problems, please share with me.” Then, I ask let me know if you have been coughing for the last two weeks, experiencing loss of appetite, fever with sweating (night sweats), weight loss, or shortness of breath. If the beneficiary has been coughing for more than two weeks, we must refer them for testing. In the form, this symptom (cough for three weeks) is marked with an asterisk, and if the patient has this symptom, we will have to refer them … If the patient has other symptoms, we do not refer them...” (Medical assistant, comparison site, KII)

It implies that, enlisted sexual minority people who did not visit the DIC remained out of DIC-based TB screening by MA. As the sexual minority people often visited the DIC on a need basis, only they were screened for TB. Apart from this, there was no routine TB screening mechanism in the HIV intervention for sexual minority people. Therefore, in-person visits of sexual minority people to the DIC appeared to be a limitation for DIC-based TB screening. The DIC managers and MAs from both sites admitted to low TB screening coverage, which was also corroborated by programmatic data.

“At present there are around 900 enlisted program participants [under this DIC]. Every month around 100 program participants visit DIC for seeking healthcare services and I have screened all of them. So, in three months, I have covered around 300 persons. But the rest are out of screening! Many program participants do not come to DIC regularly but take the essential field-level services (outreach, i.e., condom, lubricant, BCC distribution, one-to-one and group educational sessions, etc.).” (Medical assistant, comparison site, KII)

### Step 2: Identifying which causal or contextual factors are malleable and have the greatest scope for change

There are diverse contextual factors within the existing TB screening approach that hinder the uptake of TB services among the participants. We presented the factors into two categories: (1) individual factors, and (2) programmatic factors.

### Individual factors

#### Low knowledge, awareness and perceived susceptibility of TB among the sexual minority people

The findings alluded to limited knowledge levels among the sexual minority people, including knowledge of TB symptoms, risk and routes of transmission, prevention and treatment strategies, etc. even though they all heard the word ‘TB’. Their lower awareness of TB was linked to individuals’ internalization of disease severity and sense of risk.

“As I do not smoke, I do not have any addiction, I am not worried about being infected with *Jokkha*’ (TB). Moreover, since no one is infected by *Jokkha* in my area, it would not affect me. I know it is a contagious (*choyache*) disease, it can spread via saliva or cough, but there is no possibility of me getting it since none of my friends have it!” (*hijra*, 55 years, Intervention site, IDI)

Their overall dearth of knowledge about TB hindered their ability to report preliminary TB symptoms (i.e., coughing for over two weeks) to the PEs or any TB service facility. In addition, low TB awareness among sexual minority people was linked to the limited dissemination of information by PEs. The IDIs indicated that available communicative materials (such as posters, billboards, handbills) were not friendly enough in ‘communicating’ with sexual minority people with limited institutional education, as these were deemed too verbose to extract essential TB-related information. At the same time, during interviews (IDIs), sexual minority people stated that they were not educated enough to read and understand or those are not in accordance with their preference.

#### Low perceived severity of TB and incomplete treatment

Findings show that their low perceived severity was influenced by a lack of understanding of the serious implications of TB, thus cultivating a nonchalant attitude towards TB. For example, a 30-year old *hijra* participant narrated her experience where she was coughing for years, was diagnosed with TB, and started taking medications from a government facility in the capital city. After taking medicines for few weeks, she felt better and perceived that her condition ameliorated and symptoms subsided. She eventually stopped taking medicines. After a few months, she started coughing and feeling severe weakness again. However, this time her TB relapsed and was treated under a category 2 treatment regimen. On the other hand, we found another *hijra* participant with a history of incomplete TB treatment, and was not interested to visit healthcare facilities for further treatment despite experiencing prolonged cough. She attributed her unwillingness to the travel costs to visit the facility, busy schedule and existing workload. At the same time, there is also an opportunity cost associated with the time invested in travelling which could have been otherwise spent working and generating income.

#### Casual attitude of sexual minority people towards health and disease

Peer educators complained that some sexual minority participants were not interested in listening to educational information as their interest merely lied in collecting condoms and lubricants. This, coupled with their nonchalant attitudes towards health, led to their distracting and inattentive nature during these sessions.

“Some of them (enlisted program participants) listen carefully; some do not pay any attention! While some of them give me time, others just say “Give me the condoms, I will leave now!” Those who work at night (sex workers) are in a hurry, and they haunt us like a ghost, “Give it to me!” They (sex workers) come into the field with ‘full preparation’. Whereas, others who are not in a hurry give us time but still do not give us any importance, rather they are distracted!” (Peer educator, comparison site, KII)

“We work with such a population, it seems that after taking behavior change communication materials from us in the evening or, at night, they (participants) feel relieved (*bechegelo*)! As they are in a hurry, they will only listen to us for five minutes but are not willing to stay for any longer without any benefit!” (DIC manager, comparison site, KII)

#### Sexual minority population’s floating nature and barriers to TB screening and testing

There are also other TB screening options available besides the DIC-based approach. Key informants expressed that under NTP, community health workers conduct household and door-to-door visits for TB screening at free of cost among the general population, regardless of their endorsement to any type. Besides, DOTS centers also make announcements on TB symptoms and testing facilities using the portable microphone in residential areas, bus stations, railway stations, and watercraft terminals during day time. Sexual minority people were not readily available in these places and had their own places to live. During IDIs, the sexual minority people mentioned their preference to keep themselves separate from the general population and often do not venture and usually not congregating into these public spheres during day time due to self-perceived stigma, fear of identity disclosure, and social exclusion. This sphere also includes the ability to avail mainstream healthcare services. In terms of NTP’s initiatives, the majority of the sexual minority people claimed during the interviews that they had never come across NTP's door-to-door TB screening and DOTS center announcements. Apart from that, many of the sexual minority people were also found to be loitering in Mazars (religious shrine/tomb), parks and other areas at night which do not align with the NTP’s screening venues and times. Moreover, due to the sexual minority people’s anticipatory fear of perceived stigma, discrimination, perceived fear of breaches in confidentiality, etc., they are not willing to visit public health centers and uptake these services, thus inherently leaving them out of TB screening coverage. This claim was corroborated by the NGOs working with NTP who claimed that they rarely come across any sexual minority people in TB screening campaigns and test centers. This also demonstrated that the number of sexual minority people visiting at public hospitals is low. From our findings, it appeared that there was higher possibility of sexual minority people to be missed from NTP’s mass screening program.

### Programmatic factors

#### Only DIC-based TB screening

In the present intervention design, MA only conducts verbal TB screening with the sexual minority people at DIC whenever they come for availing themselves of any service from the MA. The information about TB screening at the DICs is usually disseminated during the outreach sessions by the peer educators. Usually, the participants come for HIV testing services (HTS), management of sexually transmitted infections (STIs), general health services, or any other consultations with the MA. Whenever sexual minority people come for the DIC-based clinical sessions, they are entitled to verbal TB screening. However, some participants do not visit the DIC regularly, therefore some of them are not screened. As one of the MAs explained:

“I do not think, I have screened all the beneficiaries. Because, there are some beneficiaries, who come to DIC irregularly. Some beneficiaries receive services from field; they may not have any health problems, so they do not come to DIC. Or, they may have some problems, but they are ignoring it! These people are not coming to DIC for taking STI related services on a regular basis. Either they do not have any problem, or they are hiding it; and taking the medicine from outside [sources].” (MA, comparison site, KII)

#### Prioritization of HIV/STI services over TB

There was provision for delivering information related to TB along with HIV and STI among enlisted sexual minority people, both in the group and one-to-one outreach sessions. However, since the programme is mostly focused on HIV and STI, they prioritize HIV and STI over TB when disseminating information. Most of the PEs and other DIC staff have been working on HIV and STI for many years, therefore they have designed their educational sessions to mandatorily accommodate HIV and STIs in the field. However, the same scenario is not applicable to the field for TB services. Since there is no obligation to disseminate information about TB in the field, many of them are less willing to do this in the field, thus leading to sparse discussions on the field. Many of them explained that they do not feel the same magnitude of ownership towards TB compared to HIV/STI. One of them mentioned that:

“Now, there are differences between, ‘when I am given some work” and ‘when I am just working because someone told me to do so’. When you will give your child to me for rearing, I will be affectionate only for a while. But I will love my own child endlessly. Till now, we do not own TB [as our work]. We were asked to deliver TB-related messages, but if we actually owned it, you would get many referral cases. We screened so many participants, but there is no TB positive participant!” (Outreach Supervisor, Comparison site, KII)

As a result, TB is rarely discussed in the field with the sexual minority people. One of the PEs mentioned that:

“About TB, we do not discuss regularly, we explain [*about TB*] occasionally (*majhe moddhay*). If we find any of the programme participants coughing, or, if anyone reports that he is suffering from cough, then we discuss TB. To tell the truth, we do not discuss TB every week; we discuss it once or twice a month. All the time, we work on HIV.” (Peer Educator, Intervention site, KII)

The current HIV prevention program followed monthly and quarterly target-based performance on specific indicators (i.e., HIV testing services, STI management, and distribution of condom, lubricant and BCC sessions) for ensuring and evaluating the programmatic achievements. As a result, they place primary emphasis on achieving the targets of the programme. The absence of a similar target for verbal TB screening, under performance assessment indicators, challenged verbal TB screening at DIC among enlisted sexual minority people, as this activity was not emphasized to the same extent.

#### Limited capacity building initiatives on TB

Upon recruitment, PEs received extensive training under the HIV prevention program, which included a brief session on TB. Programme personnel also confirmed the paucity of specific capacity-building initiatives on TB, which was also echoed by DIC staff of different levels. We noted that, in total, twenty PEs were working at intervention and comparison DICs, and only five of them received focused training on TB organized by local NGOs working for primary healthcare and TB prevention in respective DIC catchment areas. Whereas, PEs received intermittent training on HIV and STI under the HIV prevention program. Repeated training and mandatory information discussion on HIV and STI helped the PEs to master the delivery of information dissemination sessions through constant regular practice. PEs reported that as they were not well trained and capacitated to talk about TB at outreach, they did not feel sufficiently confident to discuss TB. As one of the representatives expressed:

“From my experience, continuous capacity building at all levels is mandatory for running a service package, from peer educator to central level. The existing system lacks such initiative for the TB component. Although we have started working on TB-HIV co-infection, many of our staff do not have appropriate knowledge on TB which is a critical factor.” (Representative from NGO working on HIV prevention, KII)

“We are disseminating information on HIV from the very beginning. We are experienced/well (*paka*) aware of HIV, but the same cannot be said about TB! We have memorized all HIV information. But we become stuck (*Bajhe*), when it comes to TB! We did not get enough orientation (training) on TB, we only attended some sessions. But, the way we get hands-on training on HIV, and the HIV training manuals; to what extent are we working efficiently on HIV?” (Outreach supervisor, Comparison site, KII)

#### Working hour of DIC: barrier for employed sexual minority people

Generally, the DIC clinical sessions for STI, HTS and other health services are operated from 10 am to 4–5 pm, ranging from three to four weekdays per week. Hence, there are some sexual minority people who cannot visit clinics due to their job or other activities on a regular basis. Some of the sexual minority people have scheduled work during work days. So, they do not want to spend time coming to DIC, rather they want to invest that time in generating a sizeable income. Since their earning opportunity is limited relative to the mainstream population, when they calculate the opportunity cost of coming to DIC versus going for work, they select their work over visiting DIC. Some of them also mentioned that they are students. These people find it really difficult to visit DIC during work days for clinical services. Such preference of income over health leads to low TB screening, low TB awareness, lack of understanding on disease severity and low-risk perception. As one of the key-informants opined:

*“Hijra* collect *cholla* (money from the general public), so they do not want to visit the DIC during the day. They (*hijra*) are not interested in coming here even for an hour. This time could have been dedicated to earning money outside! Also, there are some garment workers who will not visit during the daytime. We can reach them in the evening or night, at the field, and distribute condoms and lubricants among them but they cannot come to us for clinical services in the daytime.” (Peer Educator, Intervention site, KII)

“Not all of us are unemployed. There are some students and job holders who are busy during the day time. I think it is difficult for them to come here (for the DIC clinic session). I am a student now; I am free now. This is why I am here (DIC). But when I will have classes or a job, I might not able to come regularly.” (MSW, 24 years, Intervention site, IDI)

### Step 3: Deciding how to bring about the change mechanism

All the key informants readily recommended ‘community-based TB screening’ for addressing the existing low TB screening coverage, increasing presumptive case identification and minimizing opportunities of missing TB cases amongst the sexual minority people. TB screening of sexual minority people in the community was perceived an easier pathway to reach these populations, due to their availability in the field sites, convenient timing and ability to maintain privacy:

“If there is such opportunity, they do not need to go anywhere, the screening will not be specifically noticed, their privacy is maintained, they can do it within their existing schedule, it would be better. They can share their symptoms. Then, that service provider can also disseminate knowledge, which will increase awareness. Then they can assess whether they need TB screening and they will agree to TB screening. Only then, the screening in the community will be successful and the case detection rate will increase as well, I believe!” (Physician from a government hospital for the diseases of the chest, KII).

“If they (sexual minority) can be reached at the community level, where their short history will be collected then they can be tagged with the nearest community-based facility if needed! Because they are not very interested in visiting centers like ours! … A designated representative (community peer) will be responsible for identifying the presumptive cases, and can be linked to test centers as per doctors’ advice. It will be a better approach, as he (community peer) can motivate them, and sexual minority people would be more likely to listen to him. If a peer can be capacitated for TB screening of 20 or, 50 persons, the result will be fantastic!” (Physician from a tertiary public hospital, KII).

Similarly, the majority of the sexual minority people emphasized the essence of TB screening at the community level, as resonated by the following:

“If I share my problems with you, then you will know about it! I have blood in my cough, if I cough in front of you, only then you will know about it. Otherwise, how will you understand that I have TB (*Jokkha*) symptoms? You will have to ask me, or someone who works in the field (community). Everyone will be asked individually, whether they (sexual minority people) have these symptoms?” (MSW, 35years, Comparison site, IDI).

#### Selecting actors of community-based TB screening

All groups expressed favorable opinions towards the “community people” who are known as peer educators in the current programme as the frontline actors of community-based TB screening, as a non-community person would not be familiar with the culture of sexual minority people, their attitude and behavior, and how to convince, comfort and motivate them.

Sexual minority people generally opined that non-community people may lack the same empathy of a community actor. Moreover, there might be a language barrier as sexual minority people have their own set of dialects and terminologies. Moreover, time and resource investments are required to prepare a non-community actor to serve sexual minority people (i.e., orientation with community, field dynamics, language and culture). On the other hand, since community people are well-versed with the outreach modality, professional and sexual network and residence of the enlisted sexual minority people, engaging an existing peer educator would warrant less programmatic and capacity building efforts. As some of the participants mentioned:

“[Peer educators] can do it, because they know everyone, and everybody knows them. They have a [specific] field, they will work in those areas. If a new person is recruited, he will not know or recognize anyone. If anyone sees me [at roads], who will identify that I am a *kothi*? (Interviewer: No, no–no. Even I will not understand.) Then? You will not be able to recognize me! He (peer educator) called me here, as he knows about me. A [non-community] person will not be able to do that!” (MSW, 35 years, Comparison site, IDI).

“When a new staff joins, it takes time to train them and introduce them to the service modality approaches. It takes time to grasp the idea. But there are many peer educators working with us for a long time, since the beginning of *Bandhu* (implementing NGO)! It is better to engage the experienced staff, capacitate them and assign the work, than recruit new staff, I think!” (Representative from implementing NGO, KII).

In the current DIC modality, peer educators are essentially outreach-level service providers recruited from the sexual minority community. They are usually MSM and *hijra* by demographic and aged between 18–60 years. Given their familiarity with this community, they are considered the first line of communication and the bridge between the community and the DIC services. PEs are assigned to serve a specific number of sexual minority people in different locations. These participants are reached at least once every three months (one programmatic quarter) to provide services (i.e., distribution of condoms, lubricant and BCC materials, information sharing, and referral for STI symptoms/HTS/general health services) under target-based performance. The perceived reasons and benefits of engaging existing peer educators for community-based TB screening approaches are summarized in Table 2, which covers aspects of individual factors and peer health worker-related factors.

**Table 2:** Summary of perceived reasons and benefits of engaging existing PEs for community-based TB screening for sexual minority people in urban Dhaka, Bangladesh (from January 2019 to November 2020)

Theme	Sub-theme	Quotation
Individual factors	Feeling easy to share any sensitive information with PEs, as the both belong to the same community and had good relationship and rapport	Let me give you an example, at DIC I can say, ‘I have anal problems (itchiness/infection/abscess)’. But I cannot share my problems with doctors anywhere else! Even if I am expressing it to my community peers, will I share it with my relatives or my closest ones? No! I will not be able to make them understand, what has happened to me, what is an anal problem, or why it is bleeding? But, I am sharing it with them. (*hijra*, 50 years old, FGD participant)
	Feeling comfortable, listening to their advices and internalize their advices due to closeness with PEs	Also, a non-community staff will never be able to reach them. They will never disclose their status to him, [their] gender identity, sexual orientation, nothing! But, I feel, it is important to know about gender identity, sexual orientation and marital status for risk factor assessment. But, they will not reveal it to others! [Representative from implementing NGO, KII]
	Shame to exploring gender identity and sexual orientation in front of a general (non-community) person	[Peer educators] can do it, because they know everyone, and everybody knows him. They have a [specific] field, they will work in those areas. Because, if a new person is recruited, he will not recognize anyone, he will not know about anyone. If anyone sees me [at roads], who will identify that I am a *kothi*? (Interviewer: No, no–no. Even I will not understand.) Then? You will not be able to recognize me! He (peer educator) called me here, as he knows about me. A [non-community] person will not be able to do so! (MSW, 35 years old, Comparison site, IDI)
Peer health worker related factors	Knowing about community language, attitude, behavior, how to convince and motivate as a community peer	We follow some tricks, “If you do not talk (participate), they will cease (halt) my salary. Then, he is on obligation (*Badha pore jai*)! He is taking services from me, collecting condoms and lubricants. He can, at least do it for my sake!… We know, *kothi* have soft corners, they will never want, discontinuation of one’s job or salary! They (*kothi*) wish the best for us. We plan our work following some tactics. In this way, I survived (worked) for the last 10 years! (Peer educator, intervention site, KII)
	Easy to identify and access hidden and hard to reach sexual minority people	Now, if I think about the private network of community, sexual network and professional network… At *hijra* dera, I will be able to reach my *hijra* beneficiaries and clients of *hijra*, who are my beneficiaries as well, [I can talk to] both groups! I [can] initiate our outreach [services] with full equipments there, with trained staff (peer educator), we will be able to reach maximum clients. We can take any initiative of screening [by peer educator] or, sputum collection [and testing]. (Representative from implementing NGO, KII)

### Step 4: Identifying how to deliver the change mechanism

#### Procedure of community-based verbal TB screening and referral activities

As mentioned earlier, we planned to involve the peer educators in the TB screening as part of their regular outreach activities at the field sites (spots/*dera*^1^/ residence), using a verbal TB screening form. After screening each participant, a mark will be indicated next to their name. However, peer educators were encouraged to screen sexual minority people more than once if required, depending on the symptoms.

#### Maintaining confidentiality

When interviewed, the sexual minority people were asked to reflect on the proposed TB screening process in the community. They emphasized the importance of ‘TB screening individually’ during IDIs to ensure privacy and confidentiality. Acknowledging their concern, it was planned to disseminate TB-related information in groups; yet conduct ‘one-to-one’ TB screening with consent at a ‘private’ place.

“Let’s say that I met you at *majar* (a Muslim shrine), and then you are asking me questions and suggesting something. What will happen, thousands of people will gather and notice us! I will feel uneasy then, and you cannot say everything in front of everyone. It would not be effective then.” (*hijra*, 50 years, Comparison site, IDI)

In the FGD, peer educators also agreed with the notion of confidentiality during TB screening and shared that they took verbal consent from sexual minority people before starting screening. They approved ‘individual TB screening’ for maintaining confidentiality and keeping the presumptive TB status undisclosed. A peer educator from the intervention site explained:

“First, I will take his permission, because if I suddenly start discussing, he (sexual minority) might react. ‘Brother! I will discuss *Jokkha* (TB) for two minutes with you. Are you interested?’ Then, he will either say yes or no. If he denies, I have no right to force him (...) I will try to convince him, “I am saying all these for your wellbeing. If you have *Jokkha* or, cough, please share it with me! I can help you., it [TB services] is free!” (Peer educator, intervention site, KII)

#### TB referral, clinical investigation and follow up mechanism

##### Screened negative

If the case was negative upon screening, post-screening information was provided to the sexual minority people where peer educators discussed TB symptoms, cause, TB transmission and prevention methods. They also encouraged to inform the peer educator if anyone in their vicinity presents TB symptoms or perceived progression of symptoms.

##### Screened positive

After the screening, all the presumptive cases were referred to the nearest TB test centers for clinical investigation. Along with post-screening information, they received post-screening counseling, where they were informed about the TB test procedure and the location of the nearest TB test center. During IDIs, accompanied referral was suggested by most of the sexual minority people and key informants, as they perceived it as a “confirmatory step” for ensuring the clinical investigation of presumptive cases.

##### TB test report collection

After the required test at TB test center, four possible pathways were mentioned for report collection: 1. Sexual minority people alone, 2. Sexual minority people accompanied by peer educators, 3. Peer educator alone, 4. Directly forwarding the report to DIC. Considering travel time and cost, with peer educators’ consent, it was decided that peer educators will collect the report single-handedly.

##### TB treatment and follow-up plan

The TB-positive person was linked to the nearest TB DOTS center and enrolled for treatment, so that TB treatment could be continued as per the national guideline. The team already developed a TB status disclosure form for TB positive sexual minority people, where possible names (i.e., peer educator, medical assistant and any other person they would like to inform) who was authorized to information about their TB status, would be mentioned. Signature or thumb impression of sexual minority people was taken as evidence of providing consent. Moreover, there is a follow-up mechanism where the peer educators follow-up with the participants, as well as with the staff of the DOTS center, over the phone and in-person.

Under the existing TB treatment protocol, current DOTS providers (the DOTS provider may be a facility or community-based health worker or a trained community member. They daily handover the anti-TB medicines to the TB patients and ensure the intake of drugs.) are responsible for ensuring the daily dosage of medicine of TB patients. When a person is found to be TB positive either from tests (microbiologically) or by treating physicians, s/he gets registered to the nearby DOTS centers and connected to a DOTS provider. The DOTS provider follow ups the TB patients for treatment and microscopy as per the national guideline. In facility, every day, the TB patients visit the DOTS facility and take medicine in front of the providers. In community, the DOTS providers visit the TB patients’ home on daily basis, ensure medicine intake in front of them, and document side effects if any. In our case, the DOTS provider was introduced to sexual minority TB patients and peer educators. To alleviate potential healthcare access barriers at the DOTS center, sensitization sessions were conducted with the DOTS service providers prior to the referral process.

Moreover, introducing a collaborative relationship with the peer educator helped the DOTS provider track the patient and follow up their medicine intake in person. Guardian or community peers can monitor the medicine intake of TB patients regularly. The medical assistant/DIC manager also followed up the treatment of the sexual minority people over the phone or in-person. DIC staff (peer educator or, medical assistant) maintained a follow-up registrar book for TB patients, for documenting the frequency of follow-up visits and tests, duration of treatment, health status, etc. As two of the DIC staff participants mentioned:

“Peer educators can keep a note on that [enlisted] ID [of that client] so that they can follow up regularly on different aspects like how they are feeling, whether they have medicine, and the next medicine collection date? Peer educators can advise them to visit the DOTS center.” (DIC manager, Intervention site, KII).

“There was a TB patient at my DIC. When I called him, he visited the DIC. After taking medicine for seven days, if he showed any side effects (i.e., allergy), he used to call me to ask for advice on what to do and then I strategically told him to visit the DIC. Then, I measured his blood pressure. This is a technique for giving him the satisfaction “I am getting some services from here”. He used to bring his papers and medicine strips to show me.” (Medical assistant, Intervention site, FGD participant).

### Change agents for facilitating the newly-developed intervention implementation

#### Change agent 1: Specific TB training for peer educators

Key informants identified peer educators’ focused training on TB as a prerequisite for quality assurance of TB screening in the field, the correct identification of presumptive cases, and dissemination of accurate information among sexual minority people. All peer educators at the intervention site proposed participatory training sessions (i.e., role-play, interactive discussion, field-testing) considering their limited formal schooling. The inclusion of outreach supervisors in the training was also recommended for monitoring peer educators and ascertaining their effective engagement.

“For community-based TB screening, one requirement is, [peer educators’] knowledge on [TB] and conducting [TB screening]. We often engage peer educators at outreach sites, so formal education is not mandatory here (in this case). But, for dealing with TB [and] for improving their technical knowledge, we need to consider their educational status. Most of our *hijra* (transgender) peer educators have no formal education, and MSW peer educators studied up to grade three or four. If we train them (peer educators), then they can do (TB screening) with good coordination [and efficiently]”. (Program personnel working on HIV prevention activities, KII).

#### Change agent 2: TB-related information dissemination

Considering the low educational status of the sexual minority community and upon recommendation from key informants, pictorial BCC materials (i.e., leaflets, flipcharts) were developed, which would be easy to follow and understand for any individual. Sexual minority people also perceived distributing BCC materials as a straightforward way for disseminating TB related information.

“With pictures and cartoons on paper, it is easy to understand. For example, a cartoon character, is coughing or sneezing, and how [TB] is spreading among other people. If it is explained with a picture, it will be better!” (MSW, 23 years, Intervention site, IDI).

“The leaflet is the best option as it is only one page. For example, the best approach is if a peer educator distributes a picture [of a person] with fever where it is written ‘coughs for more than two weeks’ in a larger font, explains the content and screens for TB.” (Medical assistant, Intervention site, KII).

#### Change agent 3: Sensitization of sexual minority community for eliminating anticipatory discrimination at public health facilities

Sexual minority people, specifically *hijra*, are reluctant to visit public health facilities, mainly out of fear, self-stigma and social exclusion. Their mistrust towards general population would fuel their reluctance to visit a mainstream healthcare facility. However, if they were informed about the process of TB screening and sensitized to visit the DOTS center, peer educators would be more easily able to facilitate TB screening. Key informants opined that support from influential members of the community, such as *hijra guru*, was crucial for accessing *hijra*. Most *hijra* were involved either with *cholla* (money collection from people on roads, transport and markets) or cooking at hotels/hostels as *baburchi* (cook). As sexual minority people maintain a strong network under their *guru* and abide by her advice, we planned to leverage this network/connection to access them in groups. *Guru*’s instruction to her disciples for attending scheduled TB orientation sessions was perceived as a feasible and acceptable approach to increasing access and sensitization among sexual minority people.

“*Hijra* have multiple involvements, collecting fees, cooking and other tasks! To save their time, when they gather in groups of 15-20, we try to visit them in those places on multiple occasions. We mainly communicated with *hijra guru*. Mostly, *guru* offered us to visit *dera* after we met with them. We visited the *dera* and that helped our work run smoothly.” (Program personnel, working on HIV prevention program, KII)

“Sexual minority people are inclined to mistrust general population. Hence, to motivate them, their leaders should be contacted, sensitized and counseled first. If the leaders convey that, ‘If anyone gives you proposal (for TB screening and) treatment, (please participate) you will be benefited from here, you will get a chance to know about your health. They are here to help you’. Then, *hijra* will agree, otherwise, they don’t!” (Representative from an NGO working under National TB Program, KII)

#### Change agent 4: Sensitization at TB DOTS centers

Several TB DOTS providers and NGOs work within the DIC catchment area. The key-informants recommended periodical sensitization/awareness building sessions for DOTS service providers. Such sessions would motivate the DOTS staff to cordially accept the sexual minority people. If the presumptive sexual minority cases visited the DOTS centre at last once and had at least one positive experience, they feel more confident to visit the facilities and convey their positive attitudes to their peers.

“They (sexual minority people) usually do not come to us [doctors/health facilities], so we never treat them! I have never seen this group, so we are not habituated with this issue. Organizational refresher training is arranged for service providers all the time. The staff can be counseled at training about the importance of treating and caring for them equally, free of any discrimination. Since stigma is prominent, it is important to sensitize both participants and service providers. General populations often pass comments about their dressing style, therefore healthcare providers need to be sensitized about their lifestyles!” (Public health researcher working at a research institution, KII)

Additionally, upon recommendation of several key informants, a list of existing TB screening, test and DOTS centers under the DIC catchment area was prepared, which would help peer educators to identify the nearest convenient centers for sexual minority people. The field research team collected the list of existing DOTS centers under Dhaka City Corporation, and developed the address and other particulars with the DIC manager and peer educators.

“[With a map], they (DIC staff/peer educator) will be able to track the location of [DOTS centres]. We know the locations, but some of them (peer educators) do not know. If they have a map, it would be easy to locate the nearest facility. Then, it will be advantageous for them.” (Representative from an NGO working under National TB Program, KII).

### Step 5: Testing and refining the intervention

There are some factors that hindered the tracing of some sexual minority people. Subsequently, the research team, PEs and other DIC staff have taken some initiatives and refined the intervention to optimize the search for sexual minority people. This section will delineate the initiatives taken during the intervention.

#### TB screening at home/workplace/residence of *hijra *leader or *guru *(*hijradera*)

When we found that some sexual minority people were not showing up to the spots during the outreach hours, PEs adopted the strategy of reaching out to their homes, workplaces, etc. However, some PEs mentioned that many of the sexual minority people want to avoid communication with the PE at their home because their family members are either unaware or have not accepted their sexual orientation. Therefore, they were apprehensive that the feminine attitudes of the PE would disclose their identity as an MSM. In those situations, PE took them outside their home to a convenient location for TB screening. In addition, some of the sexual minority people were contacted and screened at their workplaces as they could not regularly come to the spots due to work engagements. In those cases, PEs physically arrived at those places for TB screening. Moreover, many *hijra* did not have the freedom to visit PEs because of strict rules from the *hijra guru*. In that circumstance, to screen them, several PEs collaborated and went to those *deras* after seeking prior approval from the *guru*. In those sessions, PE collectively shared knowledge about TB and then screened them out individually.

#### TB screening at social gatherings

Concurrently, PEs also went regularly to some social gatherings locally called *majma.* Some *majmas* are street-based events where conmen sell herbal medicines. There are episodes of dancing and singing which usually entice people, including sexual minority people. PEs sometimes visited those street-based events to locate sexual minority people since they were aware that they would usually go there for entertainment and buying medicine.

#### Communication through social media/mobile phone

On many occasions, PEs used phone or other social media (e.g. imo, messenger, WhatsApp etc.) to ask them to come to spots or any other convenient locations for screening. We found during the data collection period that sexual minority people maintained a virtual community through social media. All the PEs with access to smart phones are also part of these virtual communities. When they started community-based TB screening, they added this virtual community into the process to inform their community people about this new approach and encourage them to be a part of this process. Some PEs reported that this process was useful for contacting and screening the sexual minority people. However, it is important to note that this screening is not virtual, thus warranting their physical presence. This communication tool was used to disseminate knowledge and ask the sexual minority people to meet them for TB screening purposes.

Because of these quick adaptations, PEs were able to conduct community-based TB screening with 905 sexual minority people out of 1007 listed in the DIC under the intervention. Both the sexual minority people and PE reported that they successfully screened all participants as per the guidelines given to them.

### Step 6: Collecting sufficient evidence of effectiveness to proceed to rigorous evaluation of the intervention

In general, participants from all groups (sexual minority people, peer educators, outreach supervisors and DIC managers) agreed that community-based TB screening by the peer educators was helpful for increasing verbal TB screening coverage among the target group.

The main strength of the intervention model was its ability to engage peer educators. Their existing rapport with the sexual minority people made it easier to convince them to uptake TB screening and clinical investigation, if found presumptive. Since the peer educators were able to easily interact with them in their local dialects, this facilitated their interaction and acceptance of TB screening and referral to facilities. Sexual minority people discussed their problems freely with peer educators; hence there was no scope to feel hesitant as reported by both the outreach supervisor and DIC manager.

“He (peer educator) can interact with (sexual minority) people easily… Doctors have to follow certain official decorum and interact with me in a certain way. But peer educators will not behave like doctors. He understands me, knows my needs, respects my attitudes/demands, and he will explain in a way that I will like!” (MSW, 28 years, Intervention site, IDI)

Face-to-face screening by peer educators was identified as one of the strengths of community-based TB screening, as peers observed whether sexual minority people were coughing during screening and post screening discussion. Peer educators noted down the names of ‘potential presumptive cases’ who have been coughing for the preceding seven to ten days and mobilized this list for further follow-up for TB screening. However, few sexual minority people themselves reported to peer educators for a second screening.

The accompanied referral was another strength of this peer-led intervention. When the presumptive cases were referred by peer educators, most sexual minority people hesitated to visit a public health facility due to anticipatory fear of discrimination and neglect. Secondly, the unfamiliarity of the TB testing experience, including a new location, inflicted feelings of discomfort and indecision. Thus, accompanied referrals helped ease their transition into the mainstream healthcare setting.

Information dissemination activities for increasing TB related knowledge were appreciated by most of the participants. All the interviewed sexual minority people acknowledged that they came to know about TB symptoms, causes, free testing facilities and if diagnosed, free treatment provisions from peer educators. Such information dissemination by peer educators contributed to raising awareness among the sexual minority community. Many sexual minority people reported that after getting information about TB, they were able to understand the symptoms of TB. As a result, they can now easily identify a potential presumptive case and they can gauge where they could be referred for treatment.

### Benefits of community-based TB screening model

The findings indicated some positive aspects of the intervention model, such as increased verbal TB screening, increased detection of presumptive cases and referrals, increased knowledge on TB among peer educators and sexual minority people, changed attitudes and debunked misconceptions, strict maintenance of confidentiality and enhanced post-screening counseling and information.

#### Increased verbal TB screening, presumptive cases identification and referrals

The introduction of the intervention engendered a greater number of TB screenings and presumptive case identification for sexual minority people. Since the PEs were a part of the same community, it was easier for them to situate the participants at their most frequented venues and optimize screening coverage. As a result, TB screening rate was significantly increased in the intervention site where 905 persons among 1,007 sexual minority people were screened, compared to 247 sexual minority people among 926 at the comparison site (90% vs. 27%, *p* < 0.001) within the intervention period. Because of the increased TB screenings, the rates of identification of presumptive cases also significantly increased in the intervention site relative to the comparison site (5.9% vs. 0.8%, *p* < 0.001). In addition, the referral rates were also increased in intervention site, 53 presumptive cases were found and 50 of them were successfully referred to the TB testing center and tested (3 were referred but not tested). One TB positive case was identified (2%) among 50 tested, and linked to treatment. In addition, the quantitative data for both intervention and comparison sites of pre-intervention and intervention period for TB screening, presumptive identification and testing (TB care cascade) among sexual minority people is given in Table 3.

**Table 3:** Comparison of findings between the pre-intervention and intervention period for each intervention and comparison site among sexual minority people on TB screening, presumptive identification and testing (TB care cascade)

DIC	Population size	TB screening			Presumptive case identification			TB testing		
		Pre- intervention	Intervention	*p*-value	Pre- intervention	Intervention	*p*-value	Pre- intervention	intervention	*p*-value
Intervention	1007	196	905	< 0.001	0	53	< 0.001	0	50	< 0.001
Comparison	926	195	247	0.005	1	2	NS	1	1	NS

^*^NS Non-significant

#### Increased TB-related knowledge among staff and participants

One of the aims of this intervention was to improve the knowledge of sexual minority people. Almost all IDI and FGD participants were able to accurately relay the risks, routes of transmission, symptoms and course of treatment. Many of them had some ideas about TB beforehand but those were inaccurate or incomplete. In most cases, the peer educator discussed information pertaining to TB with participants during outreach visits. Hence, because of this repeated dissemination of information, the information was engrained within their knowledge base. Some participants also reported that they felt more relieved knowing that they had provisions for free treatment and medication. Moreover, a few participants felt reassured that they had adequate knowledge to identify a presumptive case in their family or social network and attain the necessary help. Most of the PEs never had any organized and focused training session on TB before this study but received repeated and rigorous training sessions during the study. This substantially expanded their basic knowledge of TB and course of treatment. At the same time, the knowledge sharing in the field with sexual minority people and on the field monitoring and supervision helped them memorize the key messages that they were disseminating among sexual minority people.

#### Changes in attitudes towards DOTS services

The intervention model changed the participants’ attitudes towards the DOTS centre. Presumptive cases who visited DOTS centre perceived themselves as ‘confident’ to visit TB testing centre ‘alone’ in the future. They also agreed to motivate community *Gotiya* (peers) to go DOTS center, as service providers were very cordial and sympathetic to sexual minority behavior. As one of the participants mentioned:

“They have never ignored me in the DOTS center. Secondly, they have not given me any glares (*aar chokhe takai nai*). I have never faced any instances where the DOTS center people treated me harshly. Even when I went to the X ray room, where I was supposed to take off my clothes, I told him that I have a problem with being shirtless. Then he offered me a scarf (*orna*) and I placed it on my body. As they understood my situation and offered me a scarf, I feel very comfortable and pleased. I will motivate other people to go such places where I felt comfortable going.” (MSW, 35 years, Intervention site, IDI)

#### Debunking misconception about TB

The findings indicated that sexual minority people (especially *hijra*) possessed some misconceptions about TB. For example, some of the sexual minority people had the following misconceptions: “Having cough with blood is a necessary symptom of TB”, “people who do not smoke do not have any chance of catching TB”, and “TB can be infected by the saliva of an infected person”. Because of the dissemination of TB related information, several of these misconceptions were found to be dispelled both during the IDIs and when the researchers visited the field for monitoring purposes.

## Discussion

This is one of the few qualitative studies that explored the scopes of the community-based TB screening on sexual minority people. While studies from other countries have adopted similar community-based screening approaches, they were conducted on other hidden and vulnerable populations. In this discussion, our intervention model was contextualized in relation to similar models implemented in other countries. One of the prominent strengths of the model is its ability to increase the number of verbal TB screenings, detection of presumptive cases and referrals. This was also corroborated by several quantitative and qualitative studies where the community-based approach increased early case detection and notification rates, as well as referrals to more specialized healthcare centers [23–27].

Our community-based TB screening and referral intervention model was designed based on local priorities and cultural relevance; thus, adopting culturally appropriate strategies contributed to the model’s feasibility during the intervention. Throughout the development process, the sexual minority people’s engagement was invaluable, which significantly ensured the acceptability of the model among these population groups. However, the qualitative insights generated from our study also explored several technical issues, which should be considered in future program planning.

DIC-based TB screening of high-risk sexual minority groups is part of a routine HIV prevention program, aligned with the WHO recommendations [28]. However, as it was not considered as a performance indicator while evaluating HIV grant performance, thus making it a less prioritized issue. We also noted that the identification of a TB presumptive case does not necessarily guarantee completion of clinical investigation and report collection, as ‘centrally’ neither outreach supervisor nor peer educators are specifically assigned to accompany the presumptive cases. We observed that programmatic priority influences staff motivation, commitment and accountability. If presumptive case identification and referral were considered as priority activity of the intervention, this could potentially incentivize DIC staff to assume ownership over the TB component. The leadership of a DIC manager can play a crucial role in this aspect.

Poor TB knowledge among sexual minority people also suggests limited discussion on TB in the outreach modality. PEs previously did not receive any focused training on TB when they were deployed to provide HIV prevention services under the DICs, thus negatively affecting TB-related healthcare-seeking and diagnosis. We incorporated repeated information disseminations in the TB model and distributed pictorial BCC materials. This may have potentially led to increased TB knowledge among sexual minority people and they would have honed the ‘practice’ of self-reporting TB symptoms to either peer educators or the facilities.

The newly-developed TB screening model aims to identify all TB presumptive cases among enlisted sexual minority people at the intervention site, increase their TB awareness and facilitate referral and linkage to government-owned TB testing and treatment center. Previous studies have recognized TB screening and active case finding at the community level as promising approaches for early and increased TB case detection among general and marginalized populations [29–31]. In passive facility-based case finding, individuals seek TB care services due to symptoms compatible with TB. This is a “patient-initiated” strategy which limits scope of case detection in absence of patient symptom reporting [32]. In passive case-finding settings, delayed or failed linkage to care has been reported in 8–38% of TB cases [33–35].

In contrast to facility-based TB screening, community-based active case finding brings systematic TB screening and diagnosis services closer to the community and ensures service coverage of individuals who would not regularly visit health facilities [36]. In India, a simple community-based active case finding among the general population helped increase TB presumptive case identification by 87.8% and detection of sputum smear-positive TB cases by nearly 10.8% [37]. A qualitative exploration with service users and providers who were part of the community-based pulmonary TB case finding in rural Ethiopia agreed that health workers’ house-to-house visits made TB services available at the community level, and helped improved access and service utilization [23]. Such activity also contributed to an increase in the pulmonary TB case notification rate, from 64 to 127 per 100 000 population per year [23].

We observed low awareness of TB among our participants from both study sites. Although we could not find any previous study assessing TB knowledge level among sexual minority people in the country, a previous study documented poorer knowledge among community control individuals compared to the TB cases, in terms of TB knowledge on transmission, mode of transmission, knowing ≥ 1 suggestive symptom, curability of TB and availability of free treatment [38]. As many sexual minority people were not aware of TB symptoms, they did not report to the peer educators or medical assistants. Failure of presumptive cases to recognize TB symptoms at the earliest resulted in “patient’s delay” in TB care seeking [39]. Lack of TB knowledge was also attributed to critical outcomes including incomplete medication adherence, relapse cases and multidrug-resistant TB [40]. Likewise, several quantitative and qualitative studies have depicted increased knowledge and awareness about the risks and routes of transmission of TB, and its appropriate course of action [23, 41, 42]. The intervention was also found to increase knowledge among the PE and DIC staff, which is integral for ensuring a sustainable intervention, which was also reflected in community-based health workers in a similar intervention in Ethiopia [41]. One of the notable findings that were unique to our study was the ability of the community-based TB screening to debunk misconceptions about the TB testing procedure, thus eliminating the participants’ qualms about availing the services.

The sexual minority people’s lack of trust towards public health care providers could be a potential barrier to TB referral while implementing the intervention and its future scale-up. Trust and anticipated stigma were identified as key factors in influencing the sexual minority people's willingness to visit government health care facilities [9, 10]. Our findings indicated that along with regular sensitization activities, peer educators and community involvement (i.e., local community leaders and influential *guru*) are essential to create a more enabling and supportive environment for discussion and screening on TB, but also enhance community trust in the ‘public/government-operated system’. The role of community involvement is consistent with findings from previous studies [23].

Furthermore, our study findings indicated high acceptability, particularly because the focal persons were the peer educators, with whom they already have an established rapport. High rates of acceptability were also found in similar community-based interventions in different countries [23, 25, 42, 43]. A peer-led intervention based in Nepal demonstrated high success rates primarily because of the pre-existing rapports between the health workers and the participants, both of whom were People Who Inject Drugs (PWID) [42]. The findings of our study also reported that confidentiality was consistently maintained. Moreover, detailed and tailored post-screening information and counseling were provided that was well-received by the intervention participants.

It is also worth mentioning that social media has become a more prevalent mode of communication for sexual minority people therefore they were less likely to visit these spots for socializing or sex work. Since this study is also exploring facets of the sexual minority culture in Bangladesh, this insight was yet to be reflected among other populations in other studies.

### Study strengths and limitations

The study adopted a community-based approach, thus ensuring the involvement of the sexual minority people at all phases of the study to ensure the internal validity of the findings. This study developed an approach of increasing verbal TB screening for the first time among sexual minority people through an integrated model, which will harness service delivery linkages with the government health system for achieving sustainability. Since the study adopted the non-probabilistic purposive sampling approach because of the stigmatized and hidden nature of the sexual minority people, this might be subject to selection bias. However, to mitigate the chance of bias, maximum variation sampling was used [44].

## Conclusion

This study indicated that a community-based peer-led TB screening approach, including TB knowledge dissemination by capacitated peer educators and BCC sessions, could potentially enhance TB knowledge among these populations. Hence, it will be relatively inexpensive than the facility-based approach as it utilizes the existing human resources. Moreover, due to long-standing rapport, trust, and familiarity with the language, attitude and behavior of the sexual minority people, PEs might be able to influence them to uptake TB related services. Regular capacity building activities (i.e. training) on TB can be initiated for the PEs to ensure quality TB screening and knowledge dissemination at the outreach. Simple, easily understandable, user friendly and pictorial TB related BCC materials need to be introduced considering the limited education of the sexual minority people. Therefore, this study recommends this approach could be incorporated to complement the existing TB program for TB screening and information dissemination on TB among these groups of population. In achieving the target of “leaving no one behind”, leveraging such innovations in health service delivery, particularly for these stigmatized and vulnerable populations will help minimize inequities and disparities in TB case detection, thus facilitating the achievement of Universal Health Coverage by 2030. Therefore, this study recommends that this approach should be incorporated to complement the existing TB program.

## Data Availability

The datasets generated and analyzed during the current study are not publicly available as per the data policy of icddr,b but are available from the corresponding author on reasonable request.

## Footnotes

1 The residence of the *hijra.*

## References

[1] WHO. GLOBAL TUBERCULOSIS REPORT 2022. 2022.

[2] National Tuberculosis Control Programme. Annual Report 2020. 2020.

[3] ASP. Integrated Biological and Behavioural Survey (IBBS) among Key Populations at High Risk of HIV in Bangladesh, 2020. 2021.

[4] NASP. Mapping Study and Size Estimation of Key Populations in Bangladesh for HIV Programmes 2015–2016. 2016.

[5] Bangladesh bureau of statistics. population & housing census 2022. 2022.

[6] World Health Organization (2020). UNAIDS.

[7] Stop TB Partnership. Data for action for tuberculosis: key, vulnerable and underserved populations. 2017. 2018.

[8] Willie B, Hakim AJ, Badman SG, Weikum D, Narokobi R, Coy K (2021). High prevalence of pulmonary tuberculosis among female sex workers, men who have sex with men, and transgender women in Papua New Guinea. Tropical Medicine and Health.

[9] Gourab G, Khan MNM, Hasan AR, Sarwar G, Irfan SD, Reza MM (2019). The willingness to receive sexually transmitted infection services from public healthcare facilities among key populations at risk for human immunodeficiency virus infection in Bangladesh: a qualitative study. PLoS ONE.

[10] Khan SI, Hussain MI, Parveen S, Bhuiyan MI, Gourab G, Sarker GF (2009). Living on the extreme margin: social exclusion of the transgender population (hijra) in Bangladesh. J Health Popul Nutr.

[11] UNAIDS. Ending tuberculosis and aidsa jointresponse in the era of the sustainable development goals. 2018.

[12] Golub J, Bur S, Cronin W, Gange S, Baruch N, Comstock G (2006). Delayed tuberculosis diagnosis and tuberculosis transmission. Int J Tuberc Lung Dis.

[13] UNAIDS Warns That Countries Will Miss the 2020 Target of Reducing HIV-Associated TB Deaths by 75% Unless Urgent Action Is Taken [press release]. 24 March, 2017.

[14] National Tuberculosis Control Programme. National Guideline and Operational Manual for Tuberculosis. 2021.

[15] Wight D, Wimbush E, Jepson R, Doi L (2016). Six steps in quality intervention development (6SQuID). J Epidemiol Community Health.

[16] Sarwar G, Reza M, Khan MNM, Gourab G, Rahman M, Rana AM (2020). Protocol: developing and testing community-based tuberculosis (TB) screening intervention to increase TB referral, case detection and knowledge among sexual minority people in urban Bangladesh: a mixed-method study protocol. BMJ Open.

[17] Harris AD, McGregor JC, Perencevich EN, Furuno JP, Zhu J, Peterson DE (2006). The use and interpretation of quasi-experimental studies in medical informatics. J Am Med Inform Assoc.

[18] West SG, Duan N, Pequegnat W, Gaist P, Des Jarlais DC, Holtgrave D (2008). Alternatives to the randomized controlled trial. Am J Public Health.

[19] Douglas E. Qualitative analysis: practice and innovation: Taylor & Francis; 2002.

[20] Mauthner NS, Doucet A (2003). Reflexive accounts and accounts of reflexivity in qualitative data analysis. Sociology.

[21] Gale NK, Heath G, Cameron E, Rashid S, Redwood S (2013). Using the framework method for the analysis of qualitative data in multi-disciplinary health research. BMC Med Res Methodol.

[22] Saroj Tucker GM, Ravi Kanth Mallipeddi and Parimi Prabhakar;. Improving early detection of tuberculosis among most-at-risk populations through verbal screening. India: International HIV/AIDS Alliance 2012; 2012.

[23] Yassin MA, Datiko DG, Tulloch O, Markos P, Aschalew M, Shargie EB (2013). Innovative community-based approaches doubled tuberculosis case notification and improve treatment outcome in Southern Ethiopia. PLoS ONE.

[24] Shapiro AE, Variava E, Rakgokong MH, Moodley N, Luke B, Salimi S (2012). Community-based targeted case finding for tuberculosis and HIV in household contacts of patients with tuberculosis in South Africa. Am J Respir Crit Care Med.

[25] Fatima R, Qadeer E, Enarson DA, Creswell J, Stevens R, Hinderaker SG (2014). Success of active tuberculosis case detection among high-risk groups in urban slums in Pakistan. Int J Tuberc Lung Dis.

[26] Eang MT, Satha P, Yadav RP, Morishita F, Nishikiori N, van-Maaren P (2012). Early detection of tuberculosis through community-based active case finding in Cambodia. BMC Public Health.

[27] Ssemmondo E, Mwangwa F, Kironde JL, Kwarisiima D, Clark TD, Marquez C (2016). Implementation and operational research: population-based active tuberculosis case finding during large-scale mobile HIV testing campaigns in Rural Uganda. J Acquir Immune Defic Syndr.

[28] Gilpin C, Korobitsyn A, Migliori GB, Raviglione MC, Weyer K (2018). The World Health Organization standards for tuberculosis care and management. Eur Respiratory Soc.

[29] Corbett EL, Bandason T, Duong T, Dauya E, Makamure B, Churchyard GJ (2010). Comparison of two active case-finding strategies for community-based diagnosis of symptomatic smear-positive tuberculosis and control of infectious tuberculosis in Harare, Zimbabwe (DETECTB): a cluster-randomised trial. The Lancet.

[30] Sekandi J, Neuhauser D, Smyth K, Whalen C (2009). Active case finding of undetected tuberculosis among chronic coughers in a slum setting in Kampala, Uganda. Int J Tuberc Lung Dis.

[31] Lorent N, Choun K, Thai S, Kim T, Huy S, Pe R (2014). Community-based active tuberculosis case finding in poor urban settlements of Phnom Penh, Cambodia: a feasible and effective strategy. PLoS ONE.

[32] Lönnroth K, Corbett E, Golub J, Godfrey-Faussett P, Uplekar M, Weil D (2013). Systematic screening for active tuberculosis: rationale, definitions and key considerations [State of the art series. Active case finding/screening. Number 1 in the series]. Int J Tuberc Lung Dis.

[33] Buu T, Lönnroth K, Quy H (2003). Initial defaulting in the National Tuberculosis Programme in Ho Chi Minh City, Vietnam: a survey of extent, reasons and alternative actions taken following default. Int J Tuberc Lung Dis.

[34] Botha E, Den Boon S, Verver S, Dunbar R, Lawrence K, Bosman M (2008). Initial default from tuberculosis treatment: how often does it happen and what are the reasons?. Int J Tuberc Lung Dis.

[35] De Lima YV, Evans D, Page-Shipp L, Barnard A, Sanne I, Menezes CN (2013). Linkage to care and treatment for TB and HIV among people newly diagnosed with TB or HIV-associated TB at a large, inner city South African hospital. PLoS ONE.

[36] WHO. Systematic screening for active tuberculosis: an operational guide: World Health Organization; 2015.

[37] Parija D, Patra T, Kumar A, Swain B, Satyanarayana S, Sreenivas A (2014). Impact of awareness drives and community-based active tuberculosis case finding in Odisha, India. Int J Tuberc Lung Dis.

[38] Hossain S, Zaman K, Quaiyum A, Banu S, Husain A, Islam A (2015). Factors associated with poor knowledge among adults on tuberculosis in Bangladesh: results from a nationwide survey. J Health Popul Nutr.

[39] Seid A, Metaferia Y (2018). Factors associated with treatment delay among newly diagnosed tuberculosis patients in Dessie city and surroundings, Northern Central Ethiopia: a cross-sectional study. BMC Public Health.

[40] Marahatta SB, Yadav RK, Giri D, Lama S, Rijal KR, Mishra SR (2020). Barriers in the access, diagnosis and treatment completion for tuberculosis patients in central and western Nepal: A qualitative study among patients, community members and health care workers. PLoS ONE.

[41] Yirgu R, Lemessa F, Hirpa S, Alemayehu A, Klinkenberg E (2017). Determinants of delayed care seeking for TB suggestive symptoms in Seru district, Oromiya region, Ethiopia: a community based unmatched case-control study. BMC Infect Dis.

[42] Joshi D, Sthapit R, Brouwer M (2017). Peer-led active tuberculosis case-finding among people living with HIV: lessons from Nepal. Bull World Health Organ.

[43] McDowell M, Hossain M, Rahman N, Tegenfeldt K, Yasmin N, Johnson MG (2015). Expanding tuberculosis case notification among marginalized groups in Bangladesh through peer sputum collection. Public Health Action.

[44] Khan SI, Khan MNM, Hasan AR, Irfan SD, Horng LM-S, Chowdhury EI (2019). Understanding the reasons for using methamphetamine by sexual minority people in Dhaka, Bangladesh. Int J Drug Policy.

